# Abstracts and Gleanings

**Published:** 1885-12-20

**Authors:** 


### ABSTRACTS AND GLEANINGS.

**Hypodermic Injections of Quinine in Malarial Fevers.**—A writer in the New York Medical Record says: The article in the Medical Record of July 25th, entitled "The Success of Hypodermic Injections of Quinine in Malarial Fevers," has led me to send you a brief account of my experience in the treatment of malarial fevers by this method. About a year after my arrival in India I was placed in charge of a general hospital and dispensary situated about seventy miles from Madras, in a town which, with its suburbs, contained a population of over thirty thousand inhabitants. In the year 1876 I was induced, by several articles published in the British and Indian medical journals, to try extensively the treatment of intermittent and remittent fevers by the hypodermic injection of sulphate of quinine. As over nine thousand patients were annually treated in this hospital and dispensary, I was enabled to try this treatment on a large scale.

At first I employed a solution made with ordinary sulphate of quinine and dilute hydrochloric acid, and I was astonished at the wonderful effect produced by these hypodermic injections. Cases that had been taking twenty and thirty grains per diem by the mouth without any apparent effect were cured at once by the injection of from eight to twelve grains. In the first 100 cases subjected to this treatment I had 5 cases of abscess following the injections. Not being satisfied with this result, I determined to try a solution of the quiniæ sulph. solubil. (an English preparation very similar to our bi-sulphate), prepared with a little tartaric acid. I found this a great success, I might almost say a perfect success. I have used this injection in over two thousand cases without any bad effects, with the exception of one case of small abscess. Even in this case I am inclined to think there would have been no abscess if the patient's arm had been firmly held, and if the operator had not been interrupted by the violent movenents of the chill.

During the last three years of my residence in India I was settled on the Neilgherry Hills, where I had a good opportunity of carrying on this mode of treatment by hypodermic injection, and of ascertaining that it was as efficacious with Englishmen as it had proved to be with the natives. Near the tops of these Neilgherry Hills, at an elevation of about six thousand and seven thousand feet above the level of the sea, are situated the two large sanitaria of South India, with an English population of from five thousand to ten thousand, according to the season of the year. The climate on these hills at this elevation being cool and healthy, Europeans are able to live there in comfort and bring up their families. A great number of English gentlemen are residents upon these hills, being engaged in the cultivation of coffee, tea, cinchona, or "planting," as it is termed. To work their large and valuable estates great numbers of native laborers are employed. These plantations are situated on the slopes of the hills, at an average elevation of three thousand five hundred feet, and are therefore below what is called "fever range;" that is the hilly or mountain tracts of South India are generally infected with malaria until you reach an elevation of over five thousand feet. At certain times of the year it is unsafe to sleep even for a single night on these estates. The native laborers are obliged, however, to live down there, and are therefore constantly prostrated with fever. I have frequently been called to visit one of these estates, and in a single morning would often have to administer hypodermic injections of quinine to over fifty of these native laborers. In addition to constantly and regularly employing hypodermic injections of quinine in the treatment of malarial fevers, I administered quinine in this way quite frequentlv in puerperal septicæmia, where I found it had a very beneficial effect.

A considerable number of cases of puerperal septicæmia occurred in the lying-in ward of the General Hospital and Dispensary already alluded to. The native doctors and midwives have no real knowledge of anatomy and physiology, and they often employ most violent means to hasten difficult labors. It is therefore not at all surprising that many cases of septicæmia and peritonitis result.

I have already mentioned that the solution generally was composed of quiniæ, sulph. solubil., tartaric acid, and distilled water, the strength of the solution varying from fourteen per cent. to twenty per cent. With adults I usually injected two syringefuls (that is, from five to eight decigrammes, seven to twelve grains) into the upper and outer part of the arm, or on the back of the shoulder, pushing the needle well down into the subcutaneous tissues and even into the muscle. The pain produced was always trifling and of short duration. Sometimes a little redness and very slight swelling occurred, but soon disappeared. I once administered to an English army officer, taken suddenly with a congestive chill, eight syringefuls of a sixteen per cent. solution in twelve hours, with the very best of results, except that he was somewhat deaf for a day or two. Of course, I do not mean to assert that this treatment by hypodermic injection always drove the malarial poison entirely out of the system or effected a permanent cure; but one or two injections nearly always broke up the fever and effected a cure at the time so that a patient would have no return of the fever, unless he exposed himself anew by visiting a locality where malarial fever was rife or allowed his general health to run down. I had two patients—wealthy English gentlemen—who were accustomed to come every few months and get me to give them a hypodermic injection of quinine as a prophylactic measure when they were about to visit a notoriously feverish locality. During the eighteen months that I have been practising here in Chicago, I have made use of hypodermic injections of quinine several times with perfect success. In conclusion, to show what perfect confidence I have in this mode of treatment, I have had quinine injected into my own arm on two occasions.

**Hypodermic Injection of Morphine in Uremic Convulsions.**—(S. Powell, M. D., in the British Medical Journal)

In the early part of this year I was attending a child aged six years for a slight attack of scarlet fever. At the end of a week the little patient was apparently well, though anemic. The mother was cautioned about the danger of allowing the child to be exposed in any way ; but the caution was not heeded, and the child went in and out as usual. At the end of a fortnight the mother came to me, saying the child's face was swollen, and it was very sick and cross. On visting, I found the usual train of symptoms of albuminuria, with dropsy. The skin was desquamating, and the child was exessively weak and anemic. I prescribed a purgative and an iron mixture, and ordered warm sponging and bathing. Three days after seeing the child in the above condition, I was suddenly called by its mother, as the child had been in a fit for an hour or more, and the convulsions were continuous. The child had one fit previously to this seizure, at 7 in the morning, which lasted about ten minutes. On my arrival, I found the patient in strong convulsions, perfectly insensible to all external impressions ; and it appeared certain that life could not continue long under the present conditions. Another medical man saw the case previously to my visit, and, I presume, deemed it hopeless. There was no possibility of giving medicine by the mouth ; and, not liking to trust to the slow absorption of rectal injection, I injected a solution of one-twelfth of a grain of morphine with one-hundred and twentieth of a grain of atropine under the skin of the arm. In five minutes the convulsions had entirely ceased ; the patient was sleeping quietly ; the breathing was natural, and the skin was moist and warm. There were no more fits, and the patient was soon well and able to get about again. I may add that I gave the child a vapor-bath while I was preparing the solution for injection.

**The Dangers of the Cocaine Habit.**—(Medical Record) It is asserted by the Medical and Surgical Reporter that for a long time while Peru was a Spanish colony the cultivation of coca was prohibited on account of the disastrous effects which the indulgence in it brought upon the inhabitants. We have already, since the introduction of cocaine, learned what these disastrous effects are which the systematic use of that drug induces.

And it is quite time to sound a note of warning, positive and decided, to the medical profession, regarding the administration of cocaine interally.

To some persons nothing is more fascinating than indulgence in cocaine. It relieves the sense of exhaustion, dispels mental depression, and produces a delicious sense of exhillaration and well-being. The after-effects are at first slight, almost imperceptible, but continual indulgence finally creates a craving which must be satisfied; the individual then becomes nervous, tremulous, sleepless, without appetite, and he is at last reduced to a condition of pitiable neurasthenia.

Some physicians have already fallen victims to the cocaine habit, and quite a number have observed the pernicious effects of this habit upon their patients. Dr. A. B. Shaw, of St. Louis, in the

Weekly Medical Review, relates experiences showing the effects of the substitution of the cocaine habit for that of morphine, and the evil influence of the continued use of cocaine upon the mind.

Dr. J. K. Bauduy, of St. Louis, has testified to the same effect.

Dr. Louis Bauer, of St. Louis, at the last meeting of the Mississippi Valley Medical Society, described the effects of cocaine on an inebriate. The alcohol habit was destroyed, but a cocaine habit substituted.

The patient, says Dr. Bauer, would take ten grains at one injection. The action of the cocaine ensued from fifteen to twenty-five seconds, arriving at its height in less than an hour; in two hours the effects were almost entirely gone, and reaction gradually followed. The drug had a very decided and marked effect upon the heart, the pulse rising quickly from its normal standard to one hundred and thirty beats per minute. The cocaine destroyed appetite; it also produced obstinate constipation, lasting many days and hard to overcome. The secretion of urine ceased for the time, and the sexual function was also suspended.

Dr. Bauer thinks that the cocaine habit is at least preferable to the alcohol habit. We believe that it will probably kill more quickly, if that is an advantage.

**Bihydrobromate of Quinia.**—This remedy, (Medical Age) which has only recently become known, contains 60 per cent. of pure quinine and about 30 per cent. of bromine. It is still easier soluble in water than hydrobromate. The property of all chemical unions of bromine with quinine, to become easily absorbed, to have no disagreeable side-effects, and to act as sedatives besides the specific effect of the quinine, have induced mainly French authors to prefer the bromine salts of quinine to all other salts of the latter drug. Paccoud, for instance, uses only this preparation in typhoid fever, and he believes he obtains a greater freedom from nervous symptoms in cases thus treated than is found under any other therapeutical procedure.

Dr. J. Maximowitish (Deutsche Medical Zeitung, 64, p. 727,) has also investigated the action of this quinine preparation, and he administers it in all cases where quinine is indicated and a special sedative effect is besides desirable. He says that the hydrobromate of quinine presents yellowish-white minute crystals, which are easily soluble in water. The solution of it has an acid reaction, and assumes within a few days a greenish tint, without the latter fact having any determining effect on the remedy. Its easy solubility makes this preparation specially adapted to hypodermic medication. M. employed a solution of 0.18 to 0.3 to 1.0 aq. dist. (2½ to 5 grains in 16 minims of water.) To obtain the solution as rapidly as possible, it ought to be heated and filtered. Internally the remedy is used in about the same doses, and best administered in wafers.

**Dover's Powder and its Modifications.**—(New York Medical Journal) Dr. B. W. Richardson ("Asclepiad"), after giving in brief the history of a case of "septinous pneumonia" (a term which he applies to pneumonia "induced by inhaling some toxic product from a cesspool" ), says that in cases of that sort there is no such anodyne, no such sporific febrifuge, as Dover's Powder. If he could envy any one as a therapeutist, he says, it would be the old physician who originally had the happy thought of blending astringent opium with relaxant ipecac, and both with a diuretic and laxative. He thinks it is often very good in practice to modify Dover's Powder by combining the one grain of opium and the one grain of ipecac with other salines than sulphate of potassium. True, Dover's Powder, he continues, contains the nitrates as well as the sulphate of potassium—four grains each in ten grains of the compound—and it often seems to him reasonable to revert to this form, nitrate of potassium, in small doses, being a good diuretic. He also often ventures upon other modifications : in acute rheumatic fever he usually substitutes sodium salicylate for the potassium salt; in gout, bi-carbonate of sodium; in remittent febrile cases, two grains of quinine with five sodium salicylate; and in quinsy and other febrile throat affections, chlorate of potassium.

**Creasote Water as a Local Anæsthetic.**—Dr. E. R. Squibb, in the Ephemeris for March, 1885, (Medical Age) says: The officinal aqua creasoti, or creasote water, is so important as a preparation for one special use that it is well to notice it in order to emphasize that special use. It is a simple one-per-cent. solution of wood creasote in water, and like similar solutions of carbolic acid and creasote, it is a most effective local anæsthetic, and topical dressing to burns and scalds. It is no better than the solutions of carbolic acid, or coal-tar creasote, for this purpose, but it is quite as good, so that whichever is most accessible or most convenient may be used. This creasote water, as made by the above formula—or diluted with an equal volume of water, or with more water for delicate surfaces in women and children—and applied by means of a single thickness of thin muslin, or worn-out cotton or linen, such as handkerchief stuff, and the application renewed from time to time, as the return of pain requires—it will relieve the pain of burns, and scalds in five to ten minutes, and will retain the relief as long as the applications are properly renewed, or until the painful stage is over.

It is also very effective as a local anæsthetic for general use in all painful conditions of the surface only, such as the pain of erysipelas. The benumbing effect of these phenols upon the skin is very promptly reached, and can be carried to almost any degree that is desirable, by simple management of the strengths of the solutions and the mode of application. They are true anæsthetics to the skin, while the much lauded cocaine is not.

This statement has been published so often during the past twenty years, and the treatment has been so effective in so many hands, that it is wonderful to notice how the common practice is still to us the old and comparatively useless hot dressings, such as carron oil, white lead ground in oil, flour, liniments, etc., or the newer application of solution of carbonate of sodium.

**Uses of Vinegar.**—Common domestic vinegar has some highly medicinal properties, in addition to its manifold uses in the household. It checks many forms of diarrhœa, especially mucous diarrhœa, and that attending phthisis and exhausting diseases, and is very useful in dysentery. It arrests the "stomach aches" and gripings of children. It is frequently employed with great benefit in bleeding from the lungs and stomach, and locally in uterine hemorrhage. It has arrested hemorrhage in all these conditions after ergot and other agents have failed. It stimulates the kidneys, and increases perspiration immediately after taking it, though it is recommended for the colliquative sweatings of phthisis. It often relieves obstinate hiccough. It will relieve a person who has partaken too freely of the "cup that cheers and inebriates." Drunken soldiers take vinegar or pickles to enable them to walk steadily into barracks. Locally, it is a useful application when lime has gotten into the eye. It reduces sweating when mixed with water and sponged over the body. Vinegar is often taken to reduce obesity; but its continued use in large quantities excites a catarrhal condition of the mucous coats of the stomach and intestines. Much American vinegar is made from cider, and is yellow in color; British vinegar is made from malt and unmalted grain, and is of a rich brown color; French vinegar is made from wine, the best from the light varieties, the inferior from the red; its usual color is red. It is obtained by acetous fermentation from above substances, and is an impure dilution of acetic acid. Vinegar is indicated in poisoning by caustic alkalies and lime. Radical vinegar, or glacial acetic acid, destroys warts, corns, and condylomata.

Wineglass-doses of vinegar will often promptly relieve "sick headache."—*Medical World.*

**Urethan—A New Hypnotic.**—Under the heading of "Therapeutic Notes" (in the Cincinnati Lancet and Clinic, October 17th, 1885), culled from various French and German journals, we find that Dr. R. v. Jaksch lately studied the nature and action of this new agent, with which Schmiedeberg made the first experiment upon animals and afterwards Jolly upon man, when it was found that it possesses narcotic properties. Urethan is chemically an ethylic ether of carbonic acid ($NH_2\ CO_2\ C_2\ H_5$) and consists of white crystals freely soluble in water, of a peculiar, though not unpleasant, taste and is perfectly odorless. Jaksch, after first having made a number of experiments upon animals (rabbits), by which he ascertained that urethan possesses toxic effects when given in doses of half a grain to each kilogram of the weight of the body, used this agent 110 times in twenty persons with the following result: When given in doses of one-quarter to a half gram (4 to 8 grains) no hypnotic effect was produced, but when administered in doses of one gram (16 grains) it invariably caused a sound sleep. It acts principally upon the brain, without, however, having any influence upon peripheral nerves; consequently it proved of no avail against the troublesome cough in phthsis, and the pains of neuralgia. But as it possesses no disagreeable or secondary effects, it may be given in cases where other narcotics are contraindicated as in valvula diseases or fatty degeneration of the heart, even in the most aggravated cases. The sleep produced is said to be natural and physiological, lasting until morning, and on awakening leaves no unpleasant after-effects. For this reason v. Jaksch is of the opinion that it will be of special service in the practice among children, and also for delirium tremens and other forms of mania. Urethan may be administered without any corrective, as it is almost tasteless and freely soluble, but for sensitive individuals any excipient may be added. It may be given in the form of powder or in solution.

**Hæmophilia**.—Hæmophilia is a very learned looking word, and, as it should do, it bespeaks a disease of which we know very little. The malady, which from time to time so unhappily incapacitates H. R. H. Prince Leopold, is one which most unprofessional people think to be due to some abnormal condition of the skin. A person who bleeds easily is said to have only one skin, in place of the proper number, which it must puzzle many to tell. It is not, however, any such malformation, but what it is is much less certain. Such persons bleed easily from not only the skin, when wounded, but from the gums and mouth, and mucous membranes. They also bruise easily, and in the same way it is probable that the troubles in the joints from which they suffer are to be explained by supposing some slight injury to the synovial membrane, and a subsequent escape of fluid to the cavity of the joint. We do not know what is the malformation or disease which predisposes to such an easy escape of the blood from its proper channels. The chemical constitution of the blood has been thought by some to be at fault, the smaller blood-vessels by others ; but no chemical or microscopical investigations that have been conducted as yet have been anything but contradictory, and, therefore, have been without result. One curious fact, however, has been elicited from various observations that have been made ; and this is, that it is hereditary to a marked degree, and that it is transmitted along the male much oftener than along the female line.—*British Med. Journal.*

**Successful Cerebral Surgery.**—In the Lancet, May, 1885, Dr. Macewn reports the case of a man, aged thirty-six, who in Aug., 1883, fell down stairs, and was rendered unconscious for twelve hours. In November, 1883, the patient was admitted into the Glasgow Royal Infirmary, with impairment of power in the left arm, accompanied by muscular twitchings and pricking sensations in some parts. A lesion was diagnosed in the motor cortex of the upper half of the right ascending frontal convolution, probably due to the previous injury. In Decembr the author trephined, and found a membrane-like patch over the surface of the gray matter; there was also blood effused into the substance of the brain in the ascending frontal convolution. All this was removed, the bone was replaced and after having been broken up into several small pieces, and the wound was dressed with eucalyptus gauze. The patient made a perfect recovery without a bad symptom, and two months afterward was able to do his ordinary work.

**What is Murder?**—A certain doctor was called to see a lady in the middle walks of life, she being in the third month of pregnancy, and was determined, she said, to have an abortion produced. The doctor after listening to her pathetic pleading for some little time, asked her to give her reasons. Her reply was, "Look at this child"—she was holding on her lap a little girl, aged twelve months—"not old enough to wean. *I cannot take care of two.*"

"Well," said the old doctor, "then you want to get rid of one of them, do you?"

"Yes," said the woman, in a low voice, dropping her head.

"All right," replied the doctor, with considerable firmness, and he picked up a hatchet lying near by, and quickly raised it as if to brain the little girl. The woman screamed frantically and caught the hatchet, saying, in a wild and frightened voice, "Doctor, what in the name of heaven are you going to do? Murder my little darling?"

"Yes," said the sage old doctor, "you said you wished one of them killed."

"Yes, yes, doctor, but not this one."

"But, my dear woman, I prefer to kill this one, because in the other instance I would endanger your life, probably kill you, thereby becoming a double murderer."—*Denver Medical Times.*

**Treatment of Hemorrhoids.**—Dr. H. L. Cokenower, of Iowa, (American Medical Journal) says: "A hemorrhoidal tumor being nourished by the hemorrhoidal veins, to cure it we must cut off the supply of blood to the part. This can be best done, as I have found by repeated trials, by the injection of medicine into the tumor. I have tried several formulas, but the one that I have found to excel all others is as follows:

| | | |
|---|---|---|
| ℞. | Carbolic acid | ℥ j, |
| | Creasote | gtt. xx, |
| | Olive oil, } Glycerine, } aa | ℥ j. M. |

"Unite by water bath. Inject from three to ten drops of this into the tumor. Pass needle as near base of tumor as possible without puncturing the gut, and pass the needle as near to opposite side of tumor as you can without passing through the tumor. Inject and withdraw the needle a little; then inject again, etc. In internal tumors always pass the needle horizontally with the gut. If hemorrhage occurs from puncturing tumor with needle, apply cold water with sponge and compress.

"Always reduce external tumors as soon as possible after treatment. Treat one tumor a week until all are removed.

"In fistulas, fill fistulous track with this medicine, and plug the opening. In fissures, saturate lint with compound and lay in crack.

"In treating hemorrhoidal tumors, if undue pain should occur, use laudanum and sweet oil. If patient is of constipated habits, have him use injections of warm water half an hour before going to stool. If weakness of spincter occurs, use a weak solution of sulph. zinc.

"This formula I have used for over six years, and upon at least forty different patients, without a single failure. I know what it will do when judiciously used, or I would not ask its publication.

"In reckless hands it is capable of doing irreparable harm; and any one not fully understanding the anatomy of the rectum, and the causation of hemorrhoids, should avoid its use.

"Its curative power is due to the coagulating of the blood supplying the tumor, and it atrophies instead of sloughing, as is the case with many of the popular remedies now used."

**Should Arsenic be Administered to Nursing Women?**—(N. Y. Medical Record) The question whether an infant at the breast can be poisoned by its mother's milk, when the latter has taken a large quantity of arsenic, recently came up in a medico-legal case in Paris. Dr. Brouardel was called upon to investigate a case where an infant had died suddenly, and the father was suspected some six months afterward of having attempted the mother's life. The child's body was exhumed and found completely transformed into adipocere, so that the viscera could not be separated from the other part. An analysis *en bloc* was therefore made. The body weighed six and three-fourth pounds, and contained nearly a twelfth of a grain of arsenic. It was decided that this quantity could not have been derived from the clothing or the soil. To decide in what proportions, if at all, arsenic is eliminated by the milk, Fowler's solution was administered to wet nurses in doses ranging from three to twelve drops. It was found that the milk contained a comparatively large quantity of arsenic after these doses had been taken for several days. As much as one-sixty-fourth of a grain could be obtained from about three ounces of milk. Experiments on animals, to determine the effects on the milk of one large dose, were not very conclusive, but it was shown that arsenic in these cases was constantly eliminated by the lacteal glands. The greatest care should, therefore, be exercised in giving arsenic to nursing women.

**To Remove Lime or Chemical Substances from the Eye.**—(Medical Press) No substance is so destructive as lime accidentally introduced in any quantities into the eye.

*Treatment.*—Immediately evert both lids, have the eye well syringed with water so as to wash away all particles with as little delay as possible, then wash out the eye with weak vinegar and water. If acids are introduced syringing with water affords the best chance, and afterwards a weak solution of bicarb. of soda, two or three grains to the ounce of water, will neutralize the effects of the acid.

**Swallowing a Scarf-Pin.**—(Medical and Surgical Reporter) It is well to be prepared to allay the anxiety that is always caused by the swallowing of a foreign body. Dr. Arthur Trevor reports (Lancet) the case of a baby who swallowed a spear-pointed scarf-pin about three inches long. He let matters alone, and the pin was safely passed, per rectum, in a few hours.

**Nephorotomy for Total Suppression of Urine.**—Mr. Clement Lucas performed an unique operation in Guy's Hospital, on October 20. A woman, from whom he had removed the right kidney for the total destruction of its secreting structure by large calculi and hydronephrosis, about four months ago, and who had made a rapid and complete recovery, was suddenly seized with great pain in the left kidney, followed by vomiting, headache, and suppression of urine. She passed urine last on Sunday morning, October 25, between eight and nine o'clock; and from that time till the operation on Thursday afternoon, no urine passed, and vomiting was persistent. Her medical attendant, Mr. Atkins, of Sutton, correctly interpreting the meaning of her symptoms, placed himself in communication with Mr. Lucas, and the patient was brought to London on Wednesday, October 28. It was thought that the effect of diaretics in flushing the kidney might yet be tried whilst the patient was watched. These proved of no avail, and on Thursday afternoon, the patient having become drowsy and much weaker, Mr. Lucas cut down the remaining kidney, and removed from the pelvis a conical calculus, measuring seven-eights of an inch by one-half in its greatest diameters. Total suppression had then lasted 102 hours. A free flow of urine took place at once through the wound, and the patient was then relieved of her vomiting and drowsiness. Five days after the operation she was doing well and feeling comfortable. Mr. Lucas's case of neprectomy, performed October 29, healed without suppuration or fever. The patienl sat up for the first time on the eighth day, and is now convalescent.—*British Medical Journal, October, 1885.*

**Some Uses of Cocaine in Gynecology.** —*Discussion*: Dr. Keating has used cocaine for some time in the same class of cases. He now uses 8-per cent. solutions with great success, especially in children's throats. He employs salicylate of cocaine in diptheria in a 5 or 6-per cent. solution; sensibility disappears in a short time, and he can then use any application without discomfort; he applies carbolic acid, tincture of iodine, etc., in this manner without exciting pain. He also applies cocaine before injecting carbolic acid into piles and also applies it on cotton to prevent its action ceasing too soon.

Dr. Thomas said that the strength of the solution may, with propriety, be greatly varied, and that in his practice upon the eyes even a 1-per cent. solution was strong enough to be of considerable value in conditions of irritation produced by foreign bodies in the eye; but in other cases, as urethral carbuncle, it might be well to use it even in saturated solution.

The question of strength is largely a question of expense, for in local application no toxic results are likely to be produced.—*Maryland Medical Journal.*

**For Infantile Eczema.**—(Dr. Tirera, Naples, Medical World) Thirty grains each of tar and calomel in an ounce of vaseline, is recommended as an excellent application in infantile eczema. It may be used two or three times a day.

**Salicylic Acid and Castor Oil in Psoriasis.**—Dr. Fox, of New York, (Journal of Cutaneous and Veneral Diseases) showed at a meeting of the New York Dermatological Society a girl eight years old who had psoriasis covering all the body. The patient's father and sister also has psoriasis. When she was admitted to the hospital, a two-per-cent. solution of salicylic acid in castor oil was applied to the right arm, a weak solution being used because of the great congestion of the skin. When the patient was shown the scaling was less, and many of the patches had disappeared, although the disease was spreading in other directions. To the left arm the mixture of oxide of zinc and balsam of Peru had been applied, and there was even less congestion in this situation. In the second case, the lower extremities were chiefly affected. This patient was peculiarly susceptible to the action of ammoniated mercurial ointment, even in a very small quantity exciting severe dermatitis. Chrysarobin pigment had been applied to the right leg, and a 5-per cent. solution of salicylic acid to the left leg, producing a marked improvement in the condition of the eruption in the latter situation.

**Some Lay Advice to Doctors.**—Avoid the society of your patients. Physicians should have no familiars; to be thoroughly respected, they must stand aloof from the gaze of society. A prophet has no power in his own country, neither has a physician in his own circle. Without skill it is impossible to become a flourishing physician, but without good manners all the skill of the most eminent physician will not avail you in a large capital. A good address is everything in a doctor. Never refuse a fee from any person who is able to give one, in order that you may never have occasion to take one from a man who is too poor to well afford one. It matters not how mercenary you may be accounted by the rich, so long as you are merciful to the poor. If you cannot get fees without depriving them of bread, it were better you had never been a doctor.—*Brooklyn Eagle.*

**Cocaine in Cracked Nipples.**—Dr. George C. Mecuen writes to the Boston Medical and Surgical Journal that he has found it to be a perfect anæsthetic in these cases. In three such cases a 4 per cent. solution was applied with a small brush, in five minutes a second application was made, and five minutes later two of the patients nursed directly from the nipple, the other using a nipple shield. All experienced a complete relief from pain. Furthermore it did not appear to hinder healing of the part. If time is any object a stronger solution should be used. Washing the nipple before the child nurses seems to make no difference to either mother or child.

**Menthol.**—Dr. L. Casper, after experimenting with menthol, and making experiments with it in his practice, concludes (Medical and Surgical Reporter) that menthol is an anesthetic for those sensitive nerve-terminations with which it can be brought in direct contact. It strongly excites the action of the secretory nerves.

**Chaulmoorgra Oil in Chronic Squamous Eczema.**—Dr. W. L. Chew reports (in the New Orleans Medical and Surgical Journal) a case of chronic universal squamous eczema cured by this remedy. The patient had been treated with iron, arsenic, cod-liver oil, etc., with but slight improvement. The oil was then given in two or three-drop doses, and increased to ten to fifteen drops three times a day. The best vehicle was found to be a goblet of sweetened cream. The oil was also used in the form of an ointment:

℞. Chaulmoorgra oil............................℥ ii,
Glycerene......................................ʒ iv.

To be rubbed over the body and limbs, and the cold shower-bath applied three or four times a day.

In fifteen days all the exudation had been checked ; on the nineteenth day the case was discharged cured.

**Granules of Podophyllin and Hydrastin for Headache.**—Dr. Pitts E. Howes, in a recent number of the Mass. Ec. Medical Journal, states that among the many and varied kinds of headache, and their name is legion, there is one, which seems to result from a perverted action of the liver, or rather a non-action, in which I have used with invariable success, the little pill, described by Prof. Scudder, and advertised by him in habitual constipation, viz: podophyllin, grs. 1–20; hydrastin, grs. 1–4. The action of this little pill is marvelous, and will repay for its use. The next time a case presents itself, in which you are confident that the liver is not working as it should, try these little pills and report results. I am confident that they will be favorable. If the headache comes on during the day, I usually direct that a pill be taken and the dose repeated in an hour, but at night I order two pills to be taken at bedtime.

**Chloride of Calcium.**—(Eclectic Medical Journal) Dr. R. W. Crighton (Practitioner) considers this salt of a special value in the treatment of scrofulous affections of the glands, and thinks it has an alterative action in all forms of the strumous diathesis. He uses the crystallized chloride, as the anhydrous salt forms a turbid solution and has an unpleasant taste. He gives one, two, or three grains at a dose to young children, and rarely over twelve or fifteen to adults. The dose of Coghill's solution (five ounces of the crystallized salt to twelve fluidounces of syrup) varies from five to forty minims, according to age and other circumstances. He always gives it in milk after meals.

**Cocaine in Obstinate Vomiting.**—It is said that cocaine administered in large doses internally, although it produces nausea, renders vomiting impossible. It has been tried in sea-sickness, the vomiting of pregnancy, and other cases of obstinate vomiting, with the invariable result of arresting the vomiting at once. The dose is from one-fourth to one grain. As the effects of cocaine wear off in from two to three hours, it has to be frequently repeated.—*Courier Record.*

**A New Hemostatic Agent.**—(Journal Medical de Bruxelles) Dr. Spaak claims for the following simple solution excellent, not to say fabulous results: Chloroform two parts, water one hundred parts. He says that he has used this hemostatic liquid for several months and attributes to it the following great advantages:

1. It acts with truly wonderful rapidity.
2. It possesses no escharotic action.
3. It is to be had everywhere, and may be prepared instantaneously.
4. It costs very little.
5. It possesses no disagreeable effects, and does not hinder a surgeon in his operations.

**Turpentine in Malignant Tumors.**—(North Carolina Medical Journal) Prof. Vingt, of Barcelona, employs a hypodermic injection consisting of one part of turpentine and two parts of alcohol in carcinoma and sarcoma, and has frequently succeeded (as reported in the Revista de Ciencias Medicas. No. 1, 1885) in causing these neoplasms to disappear. A local inflammation with fever lasting about eight days, was the usual consequence of the injection.

**Water as a Local Anesthetic.**—Dr. W. S. Halstead says (in the New York Medical Journal):

1. The skin can be completely anesthetized to any extent by cutaneous injections of water.
2. I have at times, of late, used water instead of cocaine in minor operations requiring skin incisions.
3. The anesthesia seldom oversteps the boundary of the bloodless wheal, but does not always vanish just as soon as hyperemia supervenes.—*Louisville Medical News.*

**Swamp Button-Bush in Rhus Poisoning.**—Dr. S. P. Hubbard, of Taunton, Mass., writes to the New York Medical Record: "As I have never seen in any medical journal the virtues of the swamp button-bush (*Cephalanthus occidentalis*) for poisons of all kinds, especially *rhus tox.*, I wish to say to the medical profession that no remedy half equals it. Try it. Make a strong tea and freely bathe the parts with it while hot.

**Gokhru.**—(Medical World) Gokhru, or Gokeroo, is the fruit of a tropical plant called pedalium murex. growing in East India. An infusion is prepared by pouring a pint of boiling water over an ounce of the fruit and macerating for two hours. It is reputed to be of great value in enuresis, seminal emissions, and impotence. The leaves are also employed for poultices; and the expressed oil is used as a condiment.

**The Sims Memorial Fund.**—(Medical Record) The cash subscriptions to the Sims Memorial Fund received by the Medical Record amount to $8,166.34, from which is deducted $406.43, for stationery, printing, postage, telegraphing, and commissions for collection. The balance, which remains on deposit with the United States Trust Company, and is subject to the order of the committee, is $7,759.91. This amount is considered sufficient to erect a suitable bronze statue to Dr. Sims, and the necessary steps to that end will be taken by the committee without further delay.

**A New Remedy for Hiccough.**—Dr. O. T. Shultz, of Mt. Vernon, Indiana, (American Practitioner) relates a case of hiccough of nine days' duration, which yielded on the morning of tenth day, to one drop of a one per cent. solution of nitro-glycerine, and repeated in one hour. The remedy was continued at intervals of two hours—gradually extended. On the twelfth day permanent relief was obtained and treatment discontinued.

**Cocaine in the Treatment of Whooping Cough.**—In a pamphlet recently published Dr. Moncorvo (Lancet, Sept. 6, '85,) has advocated the employment of cocaine as an adjunct to the treatment of the malady by resorcin, for which Dr. Moncorvo stands the sponsor. The upper parts of the larynx are first to be mopped with the one-per cent. solution of resorcin, and then a ten per cent. solution of the hydrochlorate of cocaine is applied. The cocaine should be used frequently.

**Anisic Acid.**—(Quarterly Therapeutical Review, Sept., 1885) Anisic acid is formed by the oxidation of the oil of anise. It is a new antipyretic, having antiseptic properties. It has a feeble tonic action, and increase arterial tension. Injected into the veins of animals in large doses it gives rise to epileptiform convulsions. It is also vaunted as a dressing for wounds when used in its pure state.

At the Annual session of the West Texas Medical Association, (Courier-Record) at San Antonio, October 18, 1885, the following officers were elected: President, Dr. B. E. Hadra; Vice-President, Dr. G. G. Watts; Second Vice-President, Dr. Gruebe, of New Braunfels; Secretary, Dr. D. Berry; Censors, Drs. Lowery, Herff, and Cupples. After the address of the retiring President, Dr. Tyner, the Association adjourned to partake of a grand banquet, where there was feasting and oratory up to a late hour.

**Vomiting of Pregnancy.**—(National Druggist) The latest remedy for the obstinate vomiting of pregnancy is the hydrochlorate of cocaine. Dr. Holtz (Algem. Med. Wochenschr.) says that in a case where, everything having failed, he had determined to produce abortion, but at the last moment thought of cocaine. He gave the patient 10 drops of a 3 per cent. solution, and had the satisfaction of finding the vomiting under control.

**Proprietary Remedies.**—There are said to be 5,000 patent medicines of American concoction now on the market, and the trade amounts to $20,000,000 per annum. Of this, $10,000,000 are expended in advertising, and the net profits are set down at $5,000,000.—*Druggists Circular.*

